# A critical review of household recycling barriers in the United Kingdom

**DOI:** 10.1177/0734242X211060619

**Published:** 2021-11-20

**Authors:** Saeed Oluwadipe, Hemda Garelick, Simon McCarthy, Diane Purchase

**Affiliations:** 1 Department of Natural Sciences, Faculty of Science and Technology, Middlesex University, London, UK; 2 Environment and City Management, Westminster City Council, London, UK

**Keywords:** Recycling, waste management, barriers, sustainable waste storage, waste planning, behaviours, deposit return scheme, urban environment

## Abstract

The UK recycling rate fluctuates between 45% and 47% and has consistently failed to meet the 65% target set by the post-Brexit Resource and Waste Strategy. Understanding the issues surrounding the low recycling rate in metropolitan cities in the United Kingdom will help to overcome these recycling challenges. The review examines the current situation with regard to the recycling rate and tonnage of waste produced in the United Kingdom based on available secondary waste flow data and explores different barriers related to household recycling. Many areas giving rise to the recycling challenges have been identified, including waste policy constraints, lack of effective communication, public engagement, physical barriers, service constraints, human factors and socio-economic barriers. The literature review reveals that factors such as waste policy, communication and physical factors were the most important aspects in influencing recycling rate or output. It is concluded that a multi-dimension intervention is required, which includes a thorough review of waste policy, a more stringent enforcement, an improved communication strategy and a more integrated planning development policy to mitigate issues affecting the United Kingdom’s low recycling rate or output. This approach will propel the local authorities to launch or initiate effective recycling management and to put in place the required infrastructure to facilitate effective recycling activities.

## Introduction

In 2008, the European Union (EU) Waste Framework Directive (WFD) 2008/98/EC sets a recycling target of 50% for member states by 2020 (European Commission, 2020). The Waste (England and Wales) Regulations 2011 thereafter transposed the EU WFD (2008/98/EC) into law in England and Wales. The UK government has taken over the control of environmental policy from the EU after Brexit and has put in place an ambitious Resource and Waste Strategy to forge a circular economy for England. The Resource and Waste Strategy for England 2018 sets a new recycling target of 65% of municipal waste to be achieved by 2035 (Local Government Association, 2018).

The local authorities’ recycling rates are derived from the statutory waste returns submitted by all local authorities on a financial year basis. These returns are provided through the Waste Dataflow portal managed by the Department for Environment Food and Rural Affairs (DEFRA). The National Indicator (NI) 192 formula (equation (1)) (Communities and Local Government, 2007) is used to calculate the percentage of household waste sent for reuse, recycling and composting for each local authority to obtain the recycling rate league table

(1) $$\%\;\;\mathrm{recycling}=\frac{X}{Y}\times 100$$

where ***X*** is the tonnage of reuse, recycling, composting or anaerobic digestion of the household waste collected and ***Y*** is the total tonnage of household waste collected.

The X and Y values vary according to the designation of the local authority as it is a waste collection authority (WCA) or a waste disposal authority (WDA) or a unitary authority (UA).

**Figure 1.**
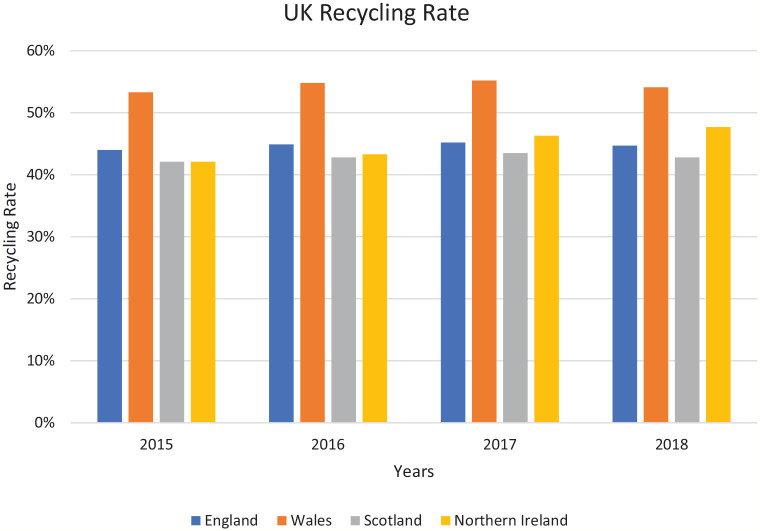
UK recycling rate from 2015 to 2018.

According to the latest waste flow data, the United Kingdom generated around 27 million tonnes per year and the recycling rate was at 46% in 2019 (DEFRA, 2020a). Household waste is collected by 408 local authorities in England, Wales, Scotland and Northern Ireland. Table 1 shows the different tonnage of waste generated from each devolved administration, and Figure 1 indicates their recycling rates. Wales has the highest recycling rate of 54% but a relatively low volume of waste. England has the highest volume of waste generated from households and Northern Ireland has the lowest volume.

**Table 1.** Waste generated from households in the United Kingdom from 2015 to 2018.

Year 2015	Devolved administration	Household waste generated in thousand tonnes	Household waste recycled in thousand tonnes
	England	22,225	9849
	Wales	1278	681
	Scotland	2354	991
	Northern Ireland	818	344
**Total**	**UK**	**26,675**	**11,865**
Year 2016	Devolved administration	Household waste generated in thousand tonnes	Household waste recycled in thousand tonnes
	England	22,770	10,217
	Wales	1307	716
	Scotland	2378	1018
	Northern Ireland	845	366
**Total**	**UK**	**27,300**	**12,318**
Year 2017	Devolved administration	Household waste generated in thousand tonnes	Household waste recycled in thousand tonnes
	England	22,437	10,139
	Wales	1271	702
	Scotland	2345	1019
	Northern Ireland	843	390
**Total**	**UK**	**26,897**	**12,250**
Year 2018	Devolved administration	Household waste generated in thousand tonnes	Household waste recycled in thousand tonnes
	England	22,033	9840
	Wales	1244	673
	Scotland	2292	981
	Northern Ireland	841	401
**Total**	**UK**	**26,411**	**11,896**

Source: DEFRA (2020a).

Overall, the UK recycling rate fluctuates between 45% and 47% and has consistently failed to meet even the lower annual recycling target of 50% of household waste set previously under the EU WFD. The data also revealed that densely populated urban boroughs (such as the City of Exeter) have relatively low recycling rates and poor performance compared to the high recycling rates for county boroughs (such as Stroud) that are sparsely populated (Table 2). Different boroughs with similar urban characteristics also present different recycling rates. For example, Newham and Bexley are both outer London boroughs and yet Bexley has the highest recycling rate and Newham has the lowest recycling rate out of all the London boroughs.

**Table 2.** England local authorities with the highest and lowest household recycling rates in each region in 2018/2019.

Region	Authority	Households recycling rate (%)	Position	Percentage of total recycling that is organic (%)	Population density (Km^2^)
London	Newham LB	17	Lowest	22	64,750
	Bexley LB	54	Highest	42	28,490
North East	Stockton-on-Tees Borough Council	26	Lowest	42	6475
	County Durham	42	Highest	31	2176
West Midlands	Birmingham City Council	22	Lowest	37	9451
	Stratford-on-Avon District Council	60	Highest	60	881
South West	Exeter City Council	27	Lowest	30	10,645
	Stroud District Council	60	Highest	42	1735
Yorkshire and the Humber	Kirklees MBC	24	Lowest	38	7200
	East Riding of Yorkshire Council	65	Highest	49	627
East Midlands	Bassetlaw District Council	25	Lowest	30	47
	South Northamptonshire District Council	60	Highest	58	1010
North West	Barrow-in-Furness Borough Council	19	Lowest	40	5698
	Cheshire West and Chester	59	Highest	48	2486
South East	Slough Borough Council	23	Lowest	42	33,670
	South Oxfordshire District Council	63	Highest	54	1399
Eastern	Tendring District Council	27	Lowest	37	2849
	Rochford District Council	63	Highest	61	3367

Source: DEFRA (2020b).

Organic materials (food and garden waste) appear to constitute a higher proportion of the recycling elements for the regions that have the highest recycling rates. It may well be possible that the county councils are facing challenges with regard to capturing recyclable materials that are non-organic. Within the London councils, such as Newham, many are struggling to recover food waste from household waste collections. Some of the local authorities, such as Westminster City Council (WCC), do not currently offer food waste collection in residential properties due to a lack of infrastructure to manage food waste storage before collection.

The recycling issue is highly complex and multifactorial. Various factors or barriers have been attributed to the causes why the target was unattainable. These phenomena could be localised and region-specific, commonly identified in most of the regions, or the results of combined effects of localised and general factors. A critical evaluation of these different barriers will enhance our understanding of the challenges and focus on resources to tackle some of the common factors. Therefore, the essence of this literature review is to reveal the different barriers and their complexity that are affecting the low recycling rate in the United Kingdom.

## Methods

This review was conducted using several databases and keywords to yield relevant literature that applies to the title of the review. A wide range of general terms and keywords that relates to the topic was initially used to search for relevant literature on several databases.

Over one hundred pieces of literature, between 1985 and 2021, including abstracts and full papers sources, were reviewed. This literature was then grouped into different categories depending on the main theme of the literature. Fifty of the studies reviewed were within the last 4 years (2017–2021), 30 sources were within the years 2010–2016, 15 sources were within the years 2000–2009 and 5 works of literature were from sources before the year 2000. In addition, secondary waste flow data were obtained from the UK government websites to interrogate relevant waste data that were used in this review.

A systematic approach was then employed to categorise the search results into the year when the article or literature was published, how relevant the literature is to the research and if the database is a recognised database for waste management. The main literature reviewed was from 2017 to 2021, to ensure that up-to-date information and trends in the waste management industry were adequately covered.

Databases such as ScienceDirect, SAGE journals, Google Scholar and the Web of Science were used to search for relevant literature. There was also limited use of Google to search for other information that was not available on databases cited above. The key terms and search words used include recycling, household recycling, household waste, deposit return scheme (DRS), recycling incentive scheme, recycling schemes in Europe, barriers to recycling, recycling behaviours, waste regulation in the United Kingdom and recycling schemes case studies.

## Results and discussion

Six categories of recycling barriers derived from literature sources based on different studies and research into recycling barriers were identified (Table 3).

**Table 3.** Types of barriers derived from different literature sources.

Barriers group	Literature sources	Comments
Physical barriers	Letelier et al. (2021); Jatau and Binbol (2020); Li et al. (2020b); Yakob et al. (2020); Díaz-Meneses and Vilkaite-Vaitone (2020); Du Toit and Wagner (2020); Rodríguez and Camilli (2018); Yukalang et al. (2017); WRAP (2014a); Timlett and Williams (2011); Jesson and Stone (2009); Barr and Gilg (2005); Ando and Gosselin (2005); Liu and Sibley (2004)	Li et al. (2020b) state that the distance to recycling facility is not a barrier.
Socio-economic barriers	Zhou et al. (2021); Mofid-Nakhaee et al. (2020); Du Toit and Wagner (2020); Knickmeyer (2020); Tsalis et al. (2018); Seng et al. (2018); Vieira and Matheus (2018); Önder (2018); Rodríguez and Camilli (2018); Yukalang et al. (2017); Dai et al. (2017); Bertoldo and Castro (2016); Becker (2014); Cole et al. (2014); Yau (2012); Prestin and Pearce (2010); Timlett and Williams (2009); Vicente and Reis (2007); Jenkins et al. (2003)	Önder (2018) asserts that income levels do not have significant impact on recycling rate. Dai et al. (2017) concluded that age factor has no substantial effect on recycling behaviours.
Human behaviours	Jatau and Binbol (2020); Rousta et al. (2020); Li et al. (2020a); Schill et al. (2020); Sung et al. (2019); Peng et al. (2018); Price (2018); Moss (2018); Eichler (2017); Institute of Leadership and Management (ILM) (2017); Watts (2017); Schill et al. (2016); Schumaker (2016); Czajkowski et al. (2015); Tabernero et al. (2015); Keighren (2015); Phipps et al. (2013); Timlett and Williams (2011); Fishbein and Ajzen (2009); Thaler and Sunstein (2008); Knussen and Yule (2008); Michie et al. (2005); Eagly and Chaiken (2005); Tonglet et al. (2004); Ajzen (1991); Bandura (1986); Ajzen (1985)	Rousta et al. (2020) concluded that human behavioural factors are the major elements that either enable or act as barriers to carrying out recycling activities.
Policy constraints	Li and Wang (2021); Ferronato et al. (2021); Sewak et al. (2021); Ayçin and Kayapinar Kaya (2021); Li et al. (2020a); DEFRA (2019, 2020c); Ogiri et al. (2019); Wiesmeth et al. (2018); Smith and Bolton (2018); HM Treasury (2018); Yukalang et al. (2017); Alfaia et al. (2017); Pollans (2017); Kirakozian (2016); Green Alliance (2014); WRAP (2014b); Cole et al. (2014); DEFRA (2012); Halvorsen (2012); European Parliament (2011); Klockner and Oppedal (2011); Abbott et al. (2011); Costa et al. (2010); DEFRA (2006); Jordan et al. (2003)	Li et al. (2020a); Halvorsen (2012) concluded that incentives, fines and penalty have weak influence on recycling habit.
Communication/public engagement	Sewak et al. (2021); Mofid-Nakhaee et al. (2020); Drimili et al. (2020); Lee (2020); Jump (2020); Lee and Krieger (2020); Al Mamun et al. (2018); Glad (2018); Satapathy (2017); Yukalang et al. (2017); WRAP (2016b); Byrne and O’Regan (2014); Miafodzyeva and Brandt (2013); De Feo and De Gisi (2010); Iyer and Kashyap (2007); Mee and Clewes (2004); Mee et al. (2004); McDonald and Oates (2003); Chan (1998).	Mofid-Nakhaee et al. (2020) indicate that public education facilitates positive influence in improving recycling quality in comparison to municipalities that do not engage in recycling public awareness.
Service/collection	Jatau and Binbol (2020); Tsalis et al. (2018); Yukalang et al. (2017); Shearer et al. (2017); Bernstad Saraiva et al. (2016); WRAP (2016a); WRAP (2016c); Sealey and Smith (2014); Timlett and Williams (2011); Entwistle (1998)	Timlett and Williams (2011) state that recycling service is one of the major factors affecting recycling rate.

### Barriers to recycling

Barriers to recycling result from a wide range of factors which could be social, physical, lack of effective community engagement, human, economic and policy constraints. Interestingly, these same factors could also be used as an intervention to implement an effective recycling system. It should be noted that all these factors are closely interwoven, and any intervention to increase the recycling rate must address all the relevant factors.

Timlett and Williams (2011) recognised three important key factors: infrastructure, service and behaviour, known as the ISB model that can be utilised to maximise recycling rates through a better understanding of the situation and context for users’ behaviours. Recent studies were undertaken by Yukalang et al. (2017); Jatau and Binbol (2020) and Du Toit and Wagner (2020) confirmed this position. It was further suggested that meaningful intervention is only possible when we understand the behaviour of the end users of products and then, to achieve a successful recycling regime, align recycling services to fit the end users’ behaviours (Timlett and Williams, 2011).

### Physical barriers

Among the top three factors of the ISB model, infrastructure is the most important in increasing the recycling rate (Du Toit and Wagner, 2020; Letelier et al., 2021; Yakob et al., 2020), especially in high-density urban areas. Waste infrastructure includes type of building, allowable internal or external storage space for waste, type of bin infrastructure, proximity to storage or recycling centres and waste collection vehicle accessibility to collect waste (Timlett and Williams, 2011).

Source segregation, another key element in achieving a high recycling rate, is wholly dependent on infrastructure. Therefore, recycling schemes with no opportunity for source segregation to occur are bound to fail (Turner et al., 2015; WRAP, 2008). The ISB model did affirm this position. In their research findings, Timlett and Williams (2011) indicated that ‘Infrastructure’ with a ‘high convenience factor’ influenced ‘Service’ to capture recyclables, which in turn initiated or triggered more positive action in resident ‘Behaviour’ than ‘Infrastructure’ with a ‘low convenience factor’ that restricted ‘Service’ to capturing recyclables.

One of the problems relating to recycling infrastructure is the non-involvement of the public in the design of the recycling infrastructure. De Feo and De Gisi (2010) suggest that recycling rates could be increased by consulting the householders in the design of waste storage infrastructure in new developments. This is justified, as these infrastructures will be utilised by the householders.

Some studies (Jatau and Binbol, 2020; Mee et al., 2004; WRAP, 2014a; Yukalang et al., 2017) have found that the common barriers to recycling are lack of space, distance to a recycling facility, inadequate infrastructure and lack of internal storage space. In terms of distance to recycling facilities, Li et al. (2020b) argued that proximity to recycling infrastructure is not a barrier to recycling practice. Their study of recycling habits in a community with similar characteristics and common factors (except for distance) found that an increased distance of 360 m to the recycling facility only has a 3% negative variation to when the distance of the recycling facility was at 80 m to the households. The distance of measurement from the households was between 80 and 360 m to the recycling facility. This assertion is in contrast to the findings of Yakob et al. (2020) and Letelier et al. (2021), both studies identified an increased distance to a recycling facility as a barrier, as residents with high travel distance to recycling infrastructure were less responsive to recycling activities compared to residents with low travel distance to recycling infrastructure. However, it is important to note that Yakob et al.’s (2020) study was conducted in a community that has different prevailing factors and situations different from the study of Li et al. (2020b), which was carried out in a community with the same factors and prevailing situations. This variance in conditions may explain the difference in the outcome of both studies.

Housing type also plays a crucial situational factor in influencing recycling intentions (Díaz-Meneses and Vilkaite-Vaitone, 2020). A resident’s intention to recycle may be obstructed by a lack of storage space, both internally and externally, to store recyclable materials. This fact was corroborated by Du Toit and Wagner (2020); their study found out that there are more recycling activities from houses compared to apartments due to the availability of storage spaces in houses and lack of spaces in flatted properties. Since the majority of buildings in the urban areas are high-rise flatted properties, in contrast to the rural areas where houses are predominant, this could be the reason why most of the local authorities with high recycling rates are located outside dense urban environments as evidenced in Table 2. In the City of Westminster, 80% of the residential housing stock are flatted properties (WCC, 2018), which indicates that the infrastructure and the types of buildings may be contributing factors to the borough’s low recycling rate. It is therefore of paramount importance that future new developments should incorporate effective waste management structures to effectively capture recyclable materials and increase recycling output.

### Socio-economic barriers

Socio-economic barriers will include population transiency, level of income, level of education, age, knowledge and awareness of environmental harm that influences human behaviour. The list is not exhaustive as the characteristics of socio-economic barriers also include factors such as homeownership, employment status, political beliefs and presence of children in the household (Becker, 2014; Knickmeyer, 2020; Vicente and Reis, 2007; Yau, 2012).

Studies have revealed that the level of education and age do affect or influence recycling outputs (Cole et al., 2014; Jenkins et al., 2003; Tsalis et al., 2018). However, Dai et al. (2017) in their study, although agreed that age is an influencing factor for recycling behaviour, argued that level of education has no substantial effect on waste behaviours of the two groups of residents and students surveyed for recycling activities. Residents with medium or high level (college or tertiary education) of education are much more aware of the environmental benefits of recycling (Prestin and Pearce, 2010; Seng et al., 2018) or can easily understand recycling communications better and therefore are in a position to respond positively to recycling campaigns or initiatives. Residents with a low level of education (no education or primary education) may not be in a position to understand the environmental benefits and therefore recycling response from this group may be low or negative coupled with other factors.

Timlett and Williams (2009) identified the impact of the transient population as one of the main factors affecting recycling behaviours in urban environments. Portsmouth City was used as a case study in the research. The study results indicate that recycling programmes in high-density housing areas associated with less transience and deprived populations are more likely to succeed than in areas with high transient and deprived populations. However, a cautionary approach has to be considered to avoid applying one recycling system to fit all localities (Knickmeyer, 2020), as individual and households’ environmental behaviours vary significantly from one locality to another (Klockner and Oppedal, 2011).

Economic factors also play a major role in affecting recycling rates. Residents in areas of deprived households may not allocate time to or focus on recycling activities because they are more preoccupied with meeting essential needs deemed more important than recycling (Knickmeyer, 2020; Smith and Bolton, 2018). A negative relationship has been found to exist between income levels and recycling rate (Önder, 2018). Seng et al. (2018), however, state that the level of income is related to the level of education and therefore greatly influences the resident’s awareness of recycling knowledge, thus resulting in positive recycling actions. This relational factor is corroborated by the study carried out by Vieira and Matheus (2018). The level of income also affects the affordability of the type of housing (Jenkins et al., 2003). Predominantly, people on low income may only afford flatted properties, which results in the low output of recycling rates, in contrast to high- or medium-income residents who can afford houses that are more convenient to accommodate effective recycling infrastructure, thereby facilitating high output recycling rate.

### Human factors

Different theories have been expounded to explain human behaviours and attitudes and how they influence response or action in a certain manner. Some researchers (Lethwaite, 1966; Michie et al., 2005; Thaler and Sunstein, 2008) have worked on theories of human behaviours, such as environmental determinism theory, behavioural change theory and the nudge theory, respectively.

The environmental determinism theory is based on the idea that the physical environment has an impact on the behaviour of people living within a specified geographical location or climatic conditions (Lethwaite, 1966). The theory has been criticised widely and rejected because of its use in justifying racial differences and imperialism (Keighren, 2015). However, the environmental determinism theory could be applied and adapted to suit certain perspectives through the application of local variables. In the recycling context, if the natural physical environment is replaced with a man-made environment (building type and type of recycling infrastructure) and the socio-cultural environment (custom, education and level of income), these replacement environments will play a role in determining individual decision-making processes (Rodríguez and Camilli, 2018) and ultimately influence their recycling behaviour.

DEFRA (2006) suggested an approach of adopting strategies and policies based on behavioural change model to influence recycling habits. This is a key shift in policy governance to move away from enforcement to the nudging approach. There are many behavioural change models and we have reviewed two major concepts: the theory of planned behaviour and the social cognitive theory (SCT).

**Figure 2.**
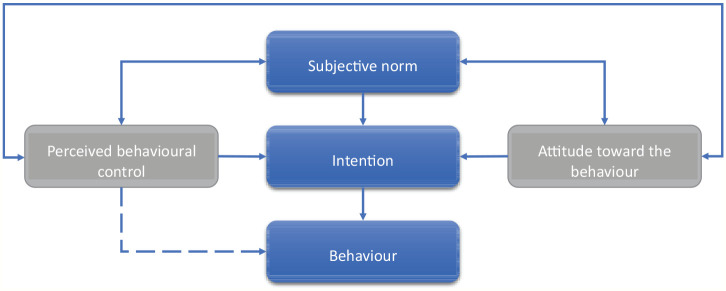
The theory of planned behaviour based on Ajzen (1991).

The theory of planned behaviour was proposed by Ajzen (1985) which describes intention as the basis of any behaviour in conjunction with other motivational factors. The more secure the intention, the higher the performance of the action (Ajzen, 1991). In this model (Figure 2), the motivational factors are attitude, subjective norm and perceived control. Attitude can be defined as hidden or concealed inclination response to physical and nonphysical objects, the response could be negative or positive depending on the nature of the inclination (Fishbein and Ajzen, 2009). Eagly and Chaiken (2005) define attitude as a speculative or theoretical configuration of the mind. Norms are societal obligations that could be formal and informal standards or rules. Norms could also be described as social pressure influencing individuals to act in a certain way. The stronger the influence, the more likely the action will be performed in the manner described by the society (Fishbein and Ajzen, 2009). Recycling studies (Byrne and O’Regan, 2014; Knickmeyer, 2020; Timlett and Williams, 2009) have shown that norms or acceptable behaviours could be localised based on the prevailing narratives in the area or peer pressure influence. A good example is ‘my neighbourhood recycles so I recycle’ or ‘my neighbourhood does not recycle so I do not recycle’.

Perceived control refers to the ability to act and self-confidence to project a successful outcome. This ability may include skills, awareness and other resources that may well include enabling and disabling factors to perform the required action (Fishbein and Ajzen, 2009). In the recycling behaviour context, a positive attitude coupled with positive societal norms and the ability to act (including enabling environment and positive intention) will result in positive recycling habits and an increase in recycling outputs (Sung et al., 2019). In contrast, a negative attitude from the inception of thought to act or not will lead to negative recycling behaviour. However, in reality, there will be other barriers or factors that may interact with the process and result in different behaviours. Rousta et al. (2020) reached the same conclusion in their study that human behavioural factors are major elements that either enable or act as barriers to carrying out recycling activities.

As an illustration, an individual may have a good attitude coupled with a positive disposition to societal norms and good intentions but lack the ability to perform the required actions (e.g. the lack of recycling infrastructure or resources to enable recycling), such individual will have no choice but to dispose of the recyclable materials as rubbish. Here, the good intentions and attitude were obstructed by external factors beyond the individual’s control.

**Figure 3.**
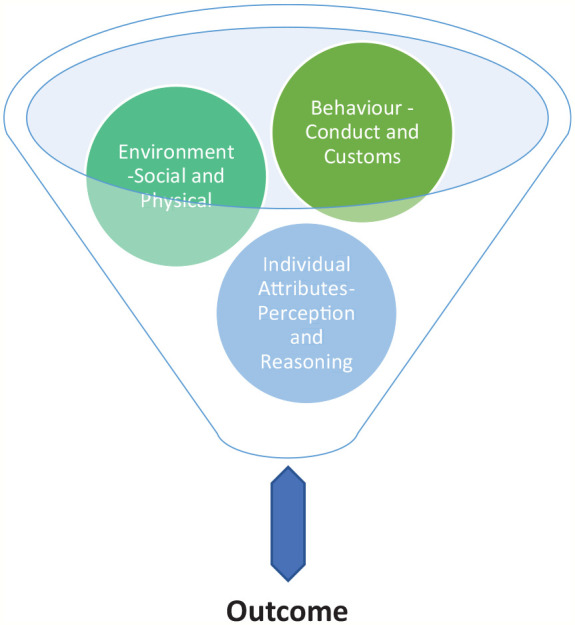
The social cognitive theory (SCT) based on Phipps et al. (2013).

The SCT (Figure 3) proposed that learning takes place in a social setting influenced by the dynamic interplay between the personal, behaviour and the environment (Bandura, 1986). In this scenario, the three factors are interconnected rather than isolated in creating an outcome. There is a need to emphasise that the ‘environment’ in SCT includes both the ‘physical and socio-cultural environment’ different from the solely ‘physical environment’ in the environmental determinism theory. SCT is very useful in understanding the dynamics and complexity underlying different elements of sustainable consumption behaviours to facilitate relevant interventions (Phipps et al., 2013), which can also be applied to understanding individual or communal recycling behaviour and the prevailing situations. Extensive works (Bertoldo and Castro, 2016; Cerda Planas, 2018; Czajkowski et al., 2015; Kirakozian, 2016; Knussen and Yule, 2008; Peng et al., 2018; Tabernero et al., 2015) have been carried out to link SCT to recycling behaviours.

Schill et al. (2020) used SCT to research children’s recycling behaviour by exposing the children to different recycling settings. The results indicate that the level of recycling participation and compliance depends on each child’s family setting, the position of the recycling point and family interaction influence. Here, the personal (knowledge), the environment (school or home) and the behaviour (past experiences) are at play in influencing different outcomes in different settings. Schill and Deirdre (2016) also found that selected interventions can be used to facilitate recycling habits. Similarly, exhibited recycling behaviours are based on attitudes, which in turn are influenced by adequate recycling awareness, accessible recycling infrastructure and not being constrained by situational factors (Tonglet et al., 2004).

Research carried out by the Institute of Leadership and Management (ILM, 2017) in 2017 shows that the younger generation (20–38 years) also known as millennials will constitute 50% of the UK workforce by 2020. It is therefore important to focus on this group to characterise their consumer behaviours.

Price (2018) detailed five characteristics of millennials with regard to the circular economy. Among these characteristics is the spending power of this age category as a prolific consumer group that will initiate greater demand for products and services specially tailored to their style and taste. Their high preference for online shopping has increased the flow of packaging waste which has necessitated the need to promote recycling education among the younger generation. Surveys carried out in the United Kingdom have indicated different recycling behaviours for the millennials. A poll of 3000 respondents carried out in 2017 found out that 49% of the age group 16–34 years always recycle compared to 70% of the age group 35–54 years who always recycle. The highest barrier to recycling cited by the younger population surveyed was the ambiguity in determining what materials can be recycled (Eichler, 2017).

A similar survey carried out by the waste company Veolia found that 71% of the age range 18–24 years have the opinion that the greatest responsibility to recycle lies with the local authorities compared to 58% of people over 55 years who share the same opinion (Watts, 2017). Another survey indicates that 78% of the age range 25–34 years are in the habit of recycling compared to 94% of people over the age of 55 years (Moss, 2018).

These surveys indicate that the younger generation is recycling less than the older generation. Therefore, the younger generation must be educated about the benefits of recycling, which is vital in embedding a circular economy in modern society – especially, considering that the younger generation is the future generation that will benefit most from the preservation of the environment.

### Waste policy constraints

Many studies have identified policy constraints and limitations as one of the barriers in achieving a high recycling rate in the United Kingdom even though the same policies are geared towards this objective. Li and Wang (2021) surmised that recycling schemes can only be successful when policy or decision-making tools are aligned with citizen or public behaviour. Although the United Kingdom has one of the more ambitious waste strategies to translate waste and resource management into a circular economy, these strategies lacked a robust process or system in place to achieve their objectives. Jordan et al. (2003) echoed the same concern that desired policy objectives do not always harmonise with stakeholders’ capabilities to implement the required policy ambitions.

Most waste policy interventions are devoid of coproduction in terms of understanding the user’s needs and situations and involving them in formulating strategies to resolve household recycling issues (Alfaia et al., 2017; Sewak et al., 2021). The non-involvement of citizens in formulating waste policies and strategies has resulted in public distrust in government waste policies, and thus a barrier to effective implementation of such policies (Drimili et al., 2020; Pollans, 2017). The majority of the citizens doubt whether the materials collected are genuinely recycled; many believed the materials are burned to generate electricity just like the rubbish collected, hence questioning the need to separate recyclable waste from non-recyclable waste.

Consultations carried out by DEFRA in 2012 on red tape bureaucracy with a specific theme on environmental regulation reported that stakeholders in the waste industry raise a concern about the complexity and inconsistent of 257 regulatory instruments within the UK environmental legislation framework (DEFRA, 2012). Such complexity, inconsistency and ambiguity are obstacles in delivering policy objectives (Ayçin and Kayapinar Kaya, 2021).

One of the shortcomings of waste policies and strategies in the United Kingdom is the non-recognition of adequate waste infrastructure and system to ensure source segregations of quality recyclable high-value materials for further processing into new products without recourse to virgin materials (Green Alliance, 2014). Policies are mainly directed to manufacturers, superstores, local authorities and waste companies but not to the householders who are primarily the producer of the waste. DEFRA (2019) identified that householders’ compliance is fundamental to increasing the recycling rate. This then suggests that, at the national level, there is a gap in waste policies which may aim for a holistic approach to waste management in the United Kingdom.

The issue of non-direct charging of householders for waste generated meant that local authorities rely on council tax and national government grants to run effective waste and recycling schemes. With recent national government cutbacks on funds available to local authorities, it is natural that most councils will give much credence to waste management from economic viability approach rather than to meet national recycling targets (Entwistle, 1998,). Abbott et al. (2011) also asserted that the policy which prevents local authorities in the United Kingdom to charge households directly on the amount of waste they generate is fuelling negative incentives for the majority of householders to improve their recycling habits.

Users of recycling receptacles are often confused about which material to put in correct receptacles because of a wide range of different receptacles with different colours and labels provided by the local authorities (Jesson and Stone, 2009); this situation and confusion are even more compounded if householders moved from one local authority area to another with receptacle provided in different colours and labels. This complexity and confusion stem from waste policies limitations in forging a uniform collection system among the local authorities for the whole of the United Kingdom (DEFRA, 2019). Schumaker (2016) suggested that one label is used for each material and adopted everywhere. Although it has been found that harmonising the collection system across the board may also create other problems (Knickmeyer, 2020); for example, the housing types and environmental behaviour vary in different local authority areas. Therefore, it has been argued that recycling schemes have to be tailored or modelled in line with local characteristics (Klockner and Oppedal, 2011).

The economic intervention or policy instrument to resolve the recycling problem is of two facets: the positive incentive gain (DRSs, vouchers and card points) and the negative incentive gain (fines and tax) that can be used to stimulate recycling habits in households. Mofid-Nakhaee et al. (2020) suggested that giving financial incentives to residents could promote effective recycling activities. Similarly, Zhou et al. (2021) applied the use of financial incentives to the residents where the residents see their recyclable materials as resources that they could trade to the waste collection companies for financial gain. This approach increased the recyclable waste collection by 229% in the community surveyed.

A comparison of the impact of financial penalties on the recycling rate worldwide carried out by Halvorsen (2012) found that the introduction of economic penalties resulted in negative effects. The introduction of penalties or ‘pay as you throw’ may increase incidents of waste fly-tipping or dumping in public places to avoid paying for waste disposal. In contrast, Ogiri et al. (2019) in their study of using a deterrence approach to nudge citizens to carry out recycling activities found that the introduction of negative incentives in form of fines and sanctions was a substantial factor in increasing residents’ participation in recycling activities. Similarly, the plastic bag tax introduced in the United Kingdom has cut down the rate of plastic bag usage; the latest data published by DEFRA indicate an 85–95% reduction in the use of plastic bags, in the United Kingdom, between 2018 and 2020 (DEFRA, 2020c).

In Europe, the EU Packaging Directive (94/62/EC) was the driver behind the introduction of DRS for empty drink bottles and containers. The scheme has been largely successful in increasing the recycling rates of the EU member states with mandatory DRS (European Parliament, 2011). European Parliament (2011) briefing paper on review of DRS in some European countries found that there was between 82% and 98% return rate of bottles and cans. Denmark DRS was successful in achieving an 84% recycling rate through the implementation of a mandatory DRS for drinks containers. Other EU members states, such as Germany and Estonia, also achieved a high recycling rate and return as a result of DRS implementation (European Parliament, 2011). It can therefore be concluded that any financial penalties or incentives to increase recycling need to be selective and targeted to certain recyclable materials to achieve effective implementation.

It is noteworthy that the United Kingdom is currently drafting contingency plans to implement the DRS in England (Circular, 2020). Scotland has already passed legislation to implement the scheme from July 2022 before which relevant infrastructure will be in place for the take-back scheme (Zero Waste Scotland, 2020). The scheme is also under consideration in Wales and Northern Ireland (BSDA, 2020). In introducing the DRS in the United Kingdom, Wiesmeth et al. (2018) cautioned that the scheme could only be effective if there are policy regulations that require mandatory rather than voluntary or informal deposits; in addition, such DRS must be managed, monitored and enforced by the government.

As a result of both past and current UK waste policies, the household recycling rate has increased (Abbott et al., 2011) from zero to the current 45% rate, and a shift in public behaviour and attitude towards recycling was observed. However, more work needs to be done on waste legislation to ensure future policies are formulated through stakeholders’ collaborations in aligning shared objectives to achieve effective implementation (Norris, 2019).

### Effective communication and public engagement

Recycling information and knowledge available to householders have been identified as one of the barriers to achieve a high recycling rate (Byrne and O’Regan, 2014; Lee, 2020; Miafodzyeva and Brandt, 2013). In terms of communication and resident engagement, the barriers may range from lack of public education or awareness on the benefit of recycling (Satapathy, 2017) to use of the language of instruction.

Ecoliteracy and environmental awareness play a significant role in influencing positive recycling activities of a low-income community surveyed (Al Mamun et al., 2018). This research suggested that intense public engagement can be strategically planned to target such communities to increase recycling output. Glad (2018) highlighted that the language of communication could be seen as discriminative if users or citizens within the community cannot all understand the language of communication. Therefore, the non-native English-speaking section of the community is formally excluded from recycling activities.

In the United Kingdom, in the absence of a national statutory regime, there is a variety of recycling regimes in operation. Therefore, many local authorities have taken advantage of this autonomy to introduce relevant intervention recycling schemes and collection systems to meet their national target of 50% (Cole et al., 2014) and specific local needs, such as housing types (Mühle et al., 2010) and prevailing demographical variation. A number of examples are illustrated below.

Bexley Council, a borough in Greater London Area, introduced a recycling scheme in 2011, branded as ‘London Green Points’ to nudge and engage residents to increase their recycling behaviour. Under the scheme, residents are awarded accumulated green points every time they recycle to obtain vouchers from the local authority which can be used at local retailers. Bexley Council has achieved a 54.1% recycling rate in the 2018/2019 financial year (London Data Store, 2019), which is 4% above the national target; the green point scheme has been identified as a factor in achieving this success (Jump, 2020).

The Waste and Resources Action Programme (WRAP) designed a new communication strategy for Barrow Borough Council to implement a new recycling scheme in 2008. The council wants to introduce a separate collection for cardboard and plastic and replace the existing 240-L bin with a 120-L bin for weekly collection (WRAP, 2016a). As a result of the new scheme implementation, the council achieved an increase in recycling from 22% in 2007/2008 to 36% in 2009/2010 (WRAP, 2016a).

Newcastle-under-Lyme Borough Council introduced two-phased plans to implement a new kerbside service and a fortnightly waste collection accompanied by separate weekly food waste collections. To achieve the scheme objectives, the council formed a partnership with WRAP to help improve the council communication strategy and resident engagement approach. The scheme achieved a savings of £500,000 in the year 2010/2011 and the recycling rate increased from 27% to 50% (WRAP, 2016b).

Coventry City Council introduced a new larger mixed recycling 240-L bin collection and reduced smaller bins for residual waste. WRAP helped the council to design a communication strategy to increase resident participation and the recycling rate. After the scheme was implemented, the Council made a saving of £1m and a 6% increase in recycling rate (WRAP, 2016c).

These four UK local authorities’ examples provide an insight into how different local authorities manage their recycling schemes differently as suggested by Klockner and Oppedal (2011). It also shows that majority of the UK local authorities are focussing more on communication campaigns (WRAP, 2014b) rather than carrying out in-depth studies and analyses to determine recycling behaviours. The only exception to this trend was Bexley’s Green Point scheme that focuses on behavioural change through practical residents’ involvement.

In summary, although communication has been identified as an important factor in influencing recycling, either positively through efficient recycling communication system or negatively through lack of awareness and recycling information (McDonald and Oates, 2003; WRAP, 2014b), other factors such as resident behaviours, situations, infrastructure and space also play important roles in influencing recycling rate or output (Timlett and Williams, 2011). Communication strategies employed by most local authorities in dealing with public recycling behaviour still depend on traditional approaches (Sewak et al., 2021), and therefore, there is a need to shift to contemporary methods of communication and residents’ engagement to capture a wider audience.

As evident from the review, good communication strategy plays an important role (Chan, 1998; Lee and Krieger, 2020; Mee and Clewes, 2004) in creating awareness about the UK local authorities’ recycling programmes. Local authorities could also embark on programmes, such as residents’ site tours of the recycling facilities for residents, so they can become familiar with what eventually happens to the materials collected from their households. This will dispel the recycling myth and doubts that all the materials collected are burned and there is no need to carry out source segregation. Public engagement through effective communication and organising awareness programmes to disseminate information on recycling schemes could nudge residents and householders to actively participate in recycling activities and ultimately result in a higher recycling rate.

### Service constraints

The recycling services provided to the residents by the local authorities can create conditions that are either favourable or unfavourable to the recycling activities (Timlett and Williams, 2011; Yukalang et al., 2017). Similarly, Tsalis et al. (2018) surmised that effective recycling services are an important factor in enabling a high recycling rate. This assertion was elucidated through their study where bespoke recycling services were tailored to the specific needs of different communities.

This barrier seems to be localised in certain areas, and it is situational depending on local factors such as inadequate spaces to offer additional waste streams collection (e.g. food waste) or to hold or store a large number of recyclable materials for 7 days prior to the weekly collection service. Jatau and Binbol (2020) found that collection frequency is a factor that can increase the recycling rate in urban areas’ flatted developments. Where storage space is scarce and residents rely on increased recycling collection frequency to keep up the recycling activities, these recycling materials will be lost to rubbish collection.

Less than half of councils in England (160 out of the 326) do not offer food waste collection (ITV, 2020). However, separate collection of household food waste can increase the recycling rate through a reduction in the volume of residual waste (Bernstad Saraiva et al., 2016; Sealey and Smith, 2014; Shearer et al., 2017). Therefore, local authorities with low recycling rates could benefit greatly by the introduction of borough-wide household food waste collection which can increase the borough recycling rate by at least 25%.

This assertion is evidenced from Table 2, which shows that local authorities with high recycling rates also have a high percentage of total recycling that is organic materials. For example, Stratford-on-Avon District Council recycling rate in 2019 is 60%, and the percentage of total organic that is recycling is also 60%. However, there are challenges to food waste collection, such as existing infrastructure may not be capable to support its separate collection and how food waste will be stored in flatted properties before its collection to prevent odour and rodent infestations.

## Recommendations

It is a challenging task to generalise the barriers for household recycling and one general approach would not resolve all these barriers due to specific localised conditions, prevailing situations and difficulty in predicting human behaviours. Nevertheless, the comprehensive literature review identified that the following barriers are essential to recycling in the United Kingdom: waste policy constraint, lack of effective communication /public engagement, physical barriers, service constraints, human factors and socio-economic barriers. These factors are interrelated and interdependent in most cases; when one factor is ineffective, it could result in a domino effect impacting the whole recycling system.

**Figure 4.**
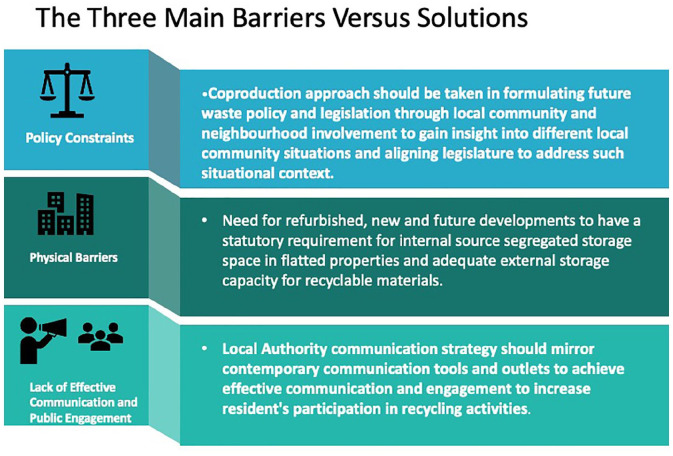
Main barriers of households recycling and potential solutions.

Out of all these barriers, the three main barriers appeared to be most impactful: the physical factors, the effectiveness of communication /public engagement employed and the influence of prevailing waste policy (Figure 4). These three main factors, therefore, need more conscientious effort in addressing the UK’s low recycling rate.

The most fundamental of all the three main causes stated above is the constraint of the available waste policy in the United Kingdom. It is fundamental as it is the bedrock of how local authorities manage and collect household waste. An effective waste policy could address all the remaining factors and will propel the local authorities to launch or initiate effective service and required infrastructure to mitigate issues affecting the United Kingdom’s low recycling output.

Furthermore, Ferronato et al. (2021) suggest the use of a selective recycling policy to target low-income communities, where neighbourhood associations in these areas can manage recycling activities to generate income for the residents and also to improve the recycling rate.

Based on these findings, the following are recommended (Figure 4):

- A co-production approach should be taken in formulating future waste policy and legislation through local community and neighbourhood involvement to gain insight into different local community situations and aligning legislature to address such situational context. Currently, no policy or regulation in England demands compulsory or mandatory recycling from householders. Therefore, the UK government should review the possibility of direct charging of residents for waste disposal to reduce the amount of waste generation and providing financial incentives to householders who recycle more of their household waste or better still make recycling a statutory or mandatory requirement on householders. This approach among other interventions will resolve the barriers associated with socio-economic factors.
- Local authorities’ communication strategy should mirror contemporary communication tools and outlets to achieve effective communication and residents’ engagement and eventually participation in recycling activities. The language of communication should be appropriate and relevant to local needs and requirements. Public engagement on recycling activities should also include the introduction of circular economy and sustainability topics in schools, colleges and universities curriculum to educate the younger generation about the benefits of recycling. Also, more importantly, to prepare the youth for future sustainable living.

## Conclusion

To achieve a high recycling rate or meet the new recycling targets of 65% set by the UK government, it is important to highlight the key barriers and address them accordingly. Of the six constraints and factors presented in this review, three have been identified as the major barriers for household recycling: physical factors, the effectiveness of communication /public engagement employed and the influence of prevailing waste policy. Therefore, a multi-dimension strategy is needed, including a thorough review of waste policy, more stringent enforcement, improved communication strategy and a more integrated development/redevelopment plan to overcome these complex and multifaceted recycling challenges.
